# Precision Angle Measurement Systems on the Basis of Ring Laser Gyro

**DOI:** 10.3390/s20236930

**Published:** 2020-12-04

**Authors:** Yuri V. Filatov, Petr A. Pavlov, Alexander A. Velikoseltsev, K. Ulrich Schreiber

**Affiliations:** 1 Department of Laser Measurement and Navigation Systems, St. Petersburg State Electrotechnical University LETI, ul. Prof. Popova 5, 197376 St. Petersburg, Russia; yvfilatov@etu.ru (Y.V.F.); papavlov@etu.ru (P.A.P.); aavelikoseltcev@etu.ru (A.A.V.); 2 Geodetic Observatory Wettzell, Research Unit Satellite Geodesy, Technical University of Munich, 93444 Bad Koetzting, Germany

**Keywords:** ring laser gyroscope, laser dynamic goniometer, angle measurements, digital angle converters, optical encoders

## Abstract

The main application of a ring laser gyroscope is navigation. It is currently the most widely used device for strapdown inertial navigation systems. However, it is also applicable for high-precision angle metrology systems. This paper discusses the properties of a laser dynamic goniometer (LDG) based on the ring laser gyroscope and designed for the calibration of optical polygons and digital angle converters, and for the measurement of angles between external mirrors (theodolite operating mode). We consider the main sources of uncertainty, such as the ring laser gyro bias due to an external magnetic field and the instability caused by the velocity of rotation along with applicable methods of their compensation. The reversal method providing separation of uncertainties of the LDG and the calibrated angle converter is analyzed in detail. The simplified cross-calibration method is also considered. The results of calibration of optical encoders of various designs—with and without their own rotors (on-axis and off-axis in Euramet terminology)—are presented. Some results of the dynamic goniometer for the measurement of angles between external mirrors are presented. It is shown that the LDG in this mode of operation demonstrates better accuracy than modern theodolites and total stations.

## 1. Introduction

**Figure 1:**
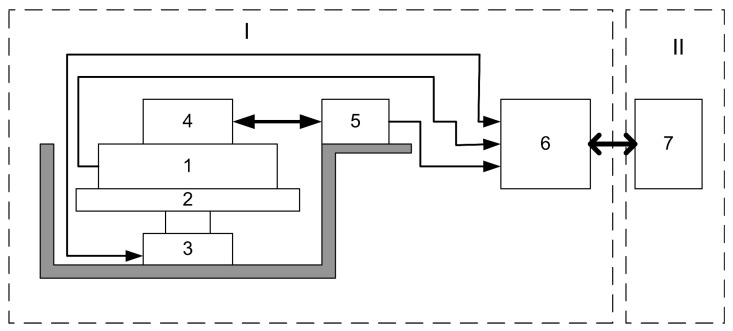
Block diagram of a dynamic laser goniometer; 1—ring laser, 2—rotary platform, 3—drive, 4—rotor, 5—stator of the angle converter, 6—electronics unit, 7—computer.

The ring laser gyro (RLG) is a well established rotation sensor for inertial navigation systems, implemented in aircraft and other moving objects [1,2]. In addition to that, it soon became clear after the very beginning of the ring laser development that the RLG is a very suitable tool for angle metrology. An angular comparator with a resolution of several thousandths of an arc second [3], based on the HEIDENHAIN radial scale, was developed by the German national metrology institution PTB some years ago, prompting other metrology institutes to achieve a similar accuracy. The preferred choice was the application of a dynamic measurement method. Therefore, the Italian Metrological Institute recently created an angular comparator with two radial scales, one of which rotates [4]. This approach allows the averaging of errors in the radial scale manufacturing and avoids interpolation errors. NIST in the USA also uses a dynamic approach based on two or sometimes three radial scales, one of which rotates [5]. Therefore, the concept of a goniometer operating in a dynamic mode, based on a HeNe ring laser gyro, has progressively come into use. This concept has been developed and evaluated by the authors for a long time [6]. This effort was renewed by a recent project, where the Pisa university, together with the Italian Metrological Institute [7], built a special ring laser in order to obtain ultra-high resolution for angular measurements in the dynamic mode. Dynamic goniometry is the part of angular metrology, where angles are measured while the test object (optical polygon, optical encoder) is rotating continuously. Tied to the test object, there is a radial scale rotating with it. In the case of a laser dynamic goniometer the radial scale is the standing wave pattern of the electromagnetic field in the cavity of the ring laser gyro. This angular scale is characterized by an extremely fine resolution and uniformity and makes up the main advantage of a dynamic ring laser goniometer. It allows the calibration of angular encoders with high precision at small angular intervals. The laser dynamic goniometer (LDG), which operates in an automated mode, is a logical extension of traditional goniometers designed to measure the angles between different faces of an optical polygon, the angular deflection of beams passing through transparent prisms and wedges, the determination of the refractive index and the dispersion of optical materials [6,8]. The LDG’s method of the angle measurement is based on the physical principle of the operation of an active Sagnac interferometer, which supports two counter-propagating electromagnetic waves in a ring cavity, whose frequency offset results from the instantaneous rate of rotation. The block diagram of the LDG is shown in Figure 1. It consists of a ring laser (1), a rotary platform (2), a drive (3), an angle converter consisting of a rotor (4) and a stator (5), an electronic unit (6) and a computer (7). As an angle converter, one can either use an angle encoder or an optical polygon with an optical null-indicator.

The block diagram presented in Figure 1 follows the terminology of [9] and is referred to as off-axis calibration. In addition to this concept, there is a whole class of dynamic goniometers in which an optical encoder is used instead of the ring laser. At the same time, this requires the calibration of the encoder; i.e., we are dealing with on-axis calibration. In this case the ring laser is also used for this purpose. In the following we consider the sources of uncertainty inherent in the laser dynamic goniometry and provide methods for reducing them.

## 2. The Main Error Sources of an LDG

The laser dynamic goniometer, and the classic goniometer based on the auto-collimation method of measurement, exhibit the following main types of measurement uncertainties: guidance uncertainty, reading uncertainty, axial system uncertainty, radial scale uncertainty and uncertainty of the controlled unit installation. The ring laser acts as a limb in the LDG. The main sources of ring laser error, which show up as a systematic uncertainty, are the influence of the external magnetic field and the instability of the rotation rate. This error shows up as a random error. In addition to that, we have the random angular walk and a random instability of the rotation rate. During the LDG operation, the periods of the ring laser output signal are summed within the time intervals formed by the output pulses of the angular converter. Any positional angle sensor can act as an angular converter, such as an optical polygon with a null-indicator, a photoelectric incremental angle encoder, a photoelectric absolute angle encoder, an inductosyn, etc. The main algorithm of the angle measurement ($\phi_{i}$) in dynamic laser goniometry is based on the principle of self-calibration, when during the measurement process, the “price” for the division of the ring laser angular scale is determined. The algorithm is defined by the expression [10]:

(1) $$\varphi_{i}=2\pi \left(\frac{N_{i}}{N_{2\pi }}\right),$$

and

(2) $$N_{i}=\frac{1}{2\pi }\int_{0}^{t_{i}}\nu \left(t\right)dt,\;\;\;\;\;\;N_{2\pi }=\frac{1}{2\pi }\int_{0}^{T_{i}}\nu \left(t\right)dt,$$

where ν(t) is the frequency of the ring laser output signal, $N_{i}$ the number of periods in the ring laser output signal falling into the time interval $t_{i}$ determined by the encoder signal and $N_{2\pi }$ the number of output signal periods during a full turn $T_{i}$. Due to fluctuations in the frequency of the output signal arising from parameter instability of the ring laser, an uncertainty in the measurement result occurs in the forming of the summation intervals, due to a minute instability of the rotation rate and other influences.

## 3. Equations for Measuring the Angle by Means of a Ring Laser

Away from the lock-in zone, the frequency of the RL output signal can be represented as [11]

(3) $$\nu \left(t\right)=K_{0}+K_{1}\omega^{*}\left(t\right)+\frac{K_{-1}}{\omega^{*}\left(t\right)}\;\;,$$

where $K_{0}$ is the bias of the ring laser output characteristic, $K_{1}$ the scale factor of the ring laser and $K_{-1}$ the non-linearity of the ring laser output characteristic. The rotation rate of the ring laser in the inertial reference frame is $\omega^{*}\left(t\right)$, which is given by $\omega^{*}\left(t\right)=\omega \left(t\right)+\Omega_{E}^{*}$. Here $\Omega_{E}^{*}$ denotes the vertical component of the angular velocity of Earth’s rotation and ω(t) the rotation rate of the ring laser in the laboratory reference frame, which in turn is rigidly connected to the LDG housing. Therefore, we have $K_{1}\omega \left(t\right)>>K_{1}\Omega_{E}^{*}>K_{0},K_{-1}/\omega^{*}\left(t\right)$. The ring laser is an absolute angular velocity sensor. Angle measurements, which determine the angle by integration, accumulate a measurement error, due to the instability of the platform rotation. By repeating the same angle in the laboratory reference frame at different periods of time, the ring laser accumulates different angles in the inertial reference frame, which results in a methodic error obtained by the measurement equation. For angular measurements using the laser dynamic method, the phase and phase-time measurement methods can be applied. In this case, they correspond to various measurement equations, which are presented in Table 1. Note that the phase method only measures the number of periods of the ring laser output signal, i.e., the phase of the ring laser signal. In the phase-time method, the time of the phase fixation is measured in addition to the phase of the ring laser signal. Table 1 also shows the methodic uncertainties corresponding to the respective measurement equations. The phase method exhibits the largest methodic error, caused by the influence of the vertical component of the Earth’s rotational velocity in the presence of an instability of the rotation rate of the turntable. Taking time into account as well allows us to eliminate this component, and the main contribution to the remaining uncertainty is the bias of the output characteristic of the ring laser (its instability). The introduction of the concept of a generalized bias $F=1/2/\pi (K_{0}+K_{1}\Omega_{E}^{*})$ allows one to reduce the methodic uncertainty of angular measurements arising from the bias of the ring laser. It is obvious that at a constant rotation rate is given, when ${\bar{\omega }}_{t}={\bar{\omega }}_{T}$ and $\varphi_{i}=\varphi_{i}^{r}$ and there is no systematic error.

## 4. Reversal Method

The main uncertainty of the LDG is due to the influence of an external magnetic field on the ring laser scale. The laser gyro has an axis of sensitivity to the magnetic field lying in the plane of the ring laser resonator [12]. During rotation, the sensitivity axis changes its orientation relative to the magnetic field lines of force, which leads to a systematic error at the first harmonic of the rotation frequency, provided that the magnetic field is uniform and constant. Let us consider the reverse method [10], which is used to separate the uncertainties of the ring laser from the encoder in order to reduce the measurement error. The calibration of the encoders on a LDG has to exclude the systematic error of the ring laser itself. This requires a separation of the systematic errors of the ring laser from the encoder (*E*). When the LDG spindle rotates “clockwise” or “counter-clockwise,” the systematic measurement error shows up as

(4) $$\begin{array}{c} \begin{array}{cc} \; & \Delta_{CW}=\Delta_{E}\left(\varphi_{i}\right)+\Delta_{RL}\left(\varphi_{i}\right)\\ \; & \Delta_{CCW}=\Delta_{E}\left(\varphi_{j}\right)+\Delta_{RL}\left(\varphi_{j}\right)\;\;, \end{array} \end{array}$$

where $\Delta_{E}\left(\varphi_{i}\right)$ and $\Delta_{E}\left(\varphi_{j}\right)$ are the encoder systematic errors for *CW* and *CCW* rotations respectively. $\Delta_{RL}\left(\varphi_{i}\right)$ and $\Delta_{RL}\left(\varphi_{j}\right)$ represent the respective systematic errors for the ring laser. $\varphi_{i}$ and $\varphi_{j}$—the angle of rotation of the LDG spindle, counted from the zero mark of the encoder, rotating in the *CW* and *CCW* directions respectively. A systematic error of the encoder shows up as the deviation of the actual value of the angle from its nominal value:

(5) $$\begin{array}{c} \begin{array}{cc} \; & \Delta_{E}\left(\varphi_{i}\right)=\varphi_{i}-i\frac{2\pi }{K}\\ \; & \Delta_{E}\left(\varphi_{j}\right)=\varphi_{j}-j\frac{2\pi }{K}\;\;, \end{array} \end{array}$$

where *K* is the number of lines on the encoder scale and is 2π/K the signal’s period. In this case the following relations are fulfilled, due to the presence of a natural standard of the angle 2π radians:

(6) $$\varphi_{i}+\varphi_{j}=2\pi$$

(7) i+j=K

From Equations (5)–(7), it follows that the systematic error of the encoder differs by the sign for the *CW* and *CCW* directions:

(8) $$\Delta_{E}\left(\varphi_{i}\right)=-\Delta_{E}\left(\varphi_{K-j}\right)$$

The systematic error of the ring laser is caused by an irregularity of its scale and affects mainly the first harmonic of the ring laser rotation frequency. When the RL rotates either counter-clockwise or clockwise, the dependence of the systematic error on the angle of rotation has the form:

(9) $$\Delta_{RL}\left(\varphi_{i},T\right)=A\left(T\right)\cos\left(\varphi_{i}+\varphi_{0}\right)$$

(10) $$\Delta_{RL}\left(\varphi_{j},T\right)=A\left(T\right)\cos\left(\varphi_{j}+\varphi_{0}\right)\;\;,$$

where $\Delta_{RL}\left(\varphi_{i,j},T\right)$ is the ring laser systematic error during the rotation in the *CW* and *CCW* directions respectively. *A* is the amplitude of the first harmonic of the ring laser systematic error, $\varphi_{0}$ is the initial phase of the systematic error of the ring laser and *T* the revolution period. The amplitude *A* depends on the value of the external magnetic field and the rotational velocity of the ring laser, and the initial phase $\varphi_{0}$ of the orientation of the ring laser in a magnetic field. From the Equations (6), (9) and (10), it follows that:

(11) $$\Delta_{RL}\left(\varphi_{i},T\right)=\Delta_{RL}\left(\varphi_{K-j},T\right)$$

Equation (11) illustrates that the systematic error of the ring laser does not depend on the direction of rotation, while on the contrary, the systematic error of the encoder depends on it (see (8)). These measurement properties form the basis of the reverse method. From Equation (4), we obtain expressions for the determination of the systematic error of a ring laser and the encoder, taking Equations (8) and (9) into account:

(12) $$\Delta_{RL}\left(\varphi_{i},T\right)=\frac{\Delta \left(\varphi_{i}\right)-\left(-\Delta \left(\varphi_{K-j}\right)\right)}{2}$$

(13) $$\Delta_{E}\left(\varphi_{i}\right)=\frac{\Delta \left(\varphi_{i}\right)+\left(-\Delta \left(\varphi_{K-j}\right)\right)}{2}$$

In order to eliminate the systematic error of the ring laser by the reverse method, it is necessary to:

1. Measure the angle of rotation of the LDG spindle when it rotates in either the *CW* or the *CCW* direction;
2. Find the systematic error of the LDG when rotating it in the *CW* and *CCW* direction—$\left(\Delta \left(\varphi_{i}\right),\Delta \left(\varphi_{K-j}\right)\right)$;
3. Perform the conversion $\Delta \left(\varphi_{j}\right)\to -\Delta \left(\varphi_{K-j}\right)$;
4. Find the systematic error of the encoder according to the expression (13).

## 5. Simplified Cross-Calibration Method

The classical method of cross-calibration [13] is widely used in the field of angular measurements. It enables the determination of systematic errors of the two angle converters involved in the measurements without using additional measuring tools. This method is based on successive angular displacements of one angle converter relative to another at angles determined by the resolution of the respective converter, taking into account that the systematic error for an angular displacement at an angle of 2π is zero. Let us describe the cross-calibration method for the total measured angles. The result of the measurements is a matrix of errors

(14) $$\Delta_{i,k}=\varphi_{i,k}-i\frac{2\pi }{P}=\left(b_{j}-b_{k}\right)+\left(a_{i+1}-a_{1}\right)\;\;,$$

where *i* is a row number (i=1,2,…P); *k* is the column number (k=1,2,…P); j=i+k if j≤P and j=i+k−P if j>P; and $b_{j}$ is the systematic error of the i-th mark of the converter. The systematic error of the ring laser is $a_{i}$ when it turns by the angle $\varphi_{i}$ and *P* is the number of converter marks. Solving the error matrix and by taking the ratios $\sum_{i=1}^{P}a_{i}=0$ and $\sum_{i=1}^{P}b_{i}=0$ into account, we find the errors of the converter and ring laser, which result from the obvious fact that the errors of both converters at the angle of 2π are zero:

$$-b_{i}=\frac{1}{P}\sum_{i=1}^{P}\left(\Delta_{ki}-\frac{1}{P}\sum_{k=1}^{P}\Delta_{ki}\right)$$

$$a_{i+1}=\frac{1}{P}\sum_{k=1}^{P}\left(\Delta_{ik}-\frac{1}{P}\sum_{i=1}^{P}\Delta_{ik}\right)$$

When measuring the angles of optical polygons with a low number of faces the measurement time is short and the calculation process is not particularly problematic. However, if the measurement is performed on an angular converter with a resolution of tens of seconds of arc, then the classical cross-calibration method becomes difficult to apply. When the angular displacement step of the converter exceeds the value of its granularity, the error matrix has the size P×M where *M* is the number of angular displacements. In this case the sum of systematic errors is not zero and the classical cross-calibration method does not work. For a LDG a simplified cross-calibration method can be applied, when the number of mutual angle displacements of the converter and the ring laser is significantly smaller than the number of the converter lines [14]. Let there be *M* uniform angular displacements of the converter relative to the ring laser. The number of measured angles is a multiple of the number of angular displacements (P/M=q, where *q* is an integer number). The measurement result can be written as an error matrix whose elements are

$$\Delta_{im}^{★}=\left(b_{i+j}-b_{j}\right)+\left(a_{i+1}-a_{1}\right)\;\;,$$

where j=1+q(m−1),m=1,2,…,M, i=1,2,…,P. The procedure for the error calculation of the converter can be summarized as:

1. Formation of the average across the columns in the error matrix $\Delta^{★}:\frac{1}{P}\sum_{i=1}^{P}\Delta_{im}^{★}=-b_{m}-a_{1}$.
2. Subtraction of the mean across the rows in each column: $\Delta_{im}^{★}-\frac{1}{P}\sum_{i=1}^{P}\Delta_{im}^{★}=b_{i+j}+a_{i+1}$.
3. Formation of a new error matrix ${\tilde{\Delta }}^{★}$ by shifting the rows in the columns, starting from the second downwards, so the row with the number *i* becomes the row with the number i+q(m−1). After the conversion the first row of each column in the new error matrix will contain the error of the converter $b_{2}$, the second row the error $b_{3}$, etc.
4. Averaging across rows of the error matrix ${\tilde{\Delta }}^{★}$
  (15) $$b_{i+1}=\frac{1}{M}\sum_{m=1}^{M}{\tilde{\Delta }}_{im}^{★}-\frac{1}{M}\sum_{m=1}^{M}a_{i+j}$$

Since the systematic error of the LDG is generally unknown, the calculation of the systematic error of the converter can be performed using the expression:

(16) $$b_{i+1}=\frac{1}{M}\sum_{m=1}^{M}{\tilde{\Delta }}_{im}^{★}$$

Expression (16) is the basic one in the simplified cross-calibration procedure. The use of expression (16) leads to a methodic uncertainty in the estimation of the systematic error of the converter, defined by the second term on the right side of expression (15). To estimate the value of the second term on the right side of (15) we decompose the systematic error of the LDG into a Fourier series in the range 0−2π:

$$a_{g}=\sum_{i=0}^{N-1}F_{i}^{LDG}\exp\left(i\frac{2\pi g}{N}\right)$$

*N* is the harmonic number and g=i+j. Angular displacements during the cross-calibration procedure do not change the spectrum of the systematic error of the LDG, but they change the phase. Using the shift theorem [4], we can write $F_{mi}^{LDG}=F_{1i}^{LDG}\exp\left(-i\frac{2\pi m}{M}\right)$. What follows is that the amplitude of the *i*-th harmonic at an angular displacement by an angle 2πm/M is equal to the amplitude of the *i*-th harmonic in its initial position, while the phase differs by 2πm/M radians. Averaging of the results obtained in *M* positions permits the determination of the averaged *i*-th Fourier harmonic of the systematic error of the LDG: ${\bar{F}}_{i}^{LDG}=\frac{1}{M}F_{1i}^{LDG}\sum_{m=1}^{M}\exp\left(-i\frac{2\pi m}{M}\right)$. The expression on the right side is zero for all harmonics except for those that are multiples of *M*. Thus the last term in expression (15) is determined by the sum of the *M*-th harmonic of the systematic error of the LDG and its multiples. To determine the amplitude of the *M*-th harmonic, another series of measurements with angular displacements for the angles $2\pi m/M_{1}$ must be carried out. In this case it is necessary that *M* and $M_{1}$ are not multiples of each other. Hence, the methodological uncertainty of the simplified cross-calibration procedure for uniform angular displacements of the converter on the angles 2πm/M is determined by the sum of the *M*-th harmonic of the LDG systematic error and its multiples.

## 6. Calibration of Incremental Angle Converters by Means of a Laser Dynamic Goniometer

Angle sensors are widely used in many areas of human activity. For their successful application, it is necessary to know their accuracy parameters. Therefore, much attention is paid to their calibration. Currently, there are recommendations from EURAMET [9] on the calibration of incremental angle converters (encoders), where the calibration techniques and tools are considered. However, these recommendations do not mention a laser dynamic goniometer as a calibration tool. The LDG has some advantages over traditional calibration means, such as rotary/indexing tables. In this section we consider some results of the LDG development for the purpose of the of angle converter calibration.

### 6.1. Results of Optical Encoder Calibration in the “On-Axis” Mode

**Figure 2:**
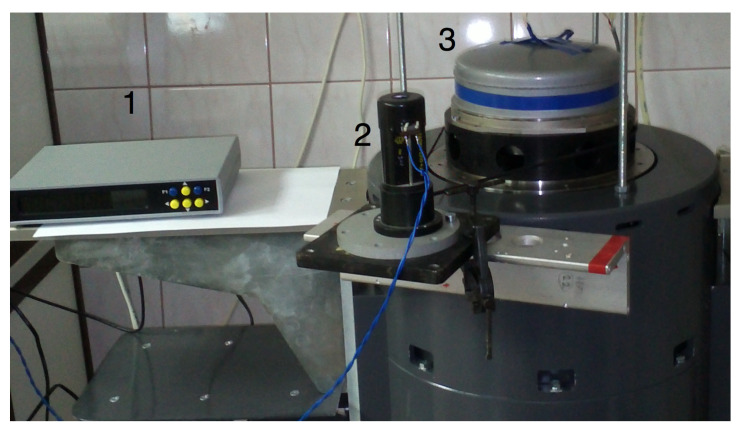
Display of the experimental laser dynamic goniometer (LDG) in the mode of an on-axis calibration tool of optical encoders: 1—optical encoder: A-205 processor, 2—DC motor, 3—ring laser.

An example of the experimental dynamic goniometer in the mode of an on-axis optical encoder calibration is shown in Figure 2.

**Figure 3:**
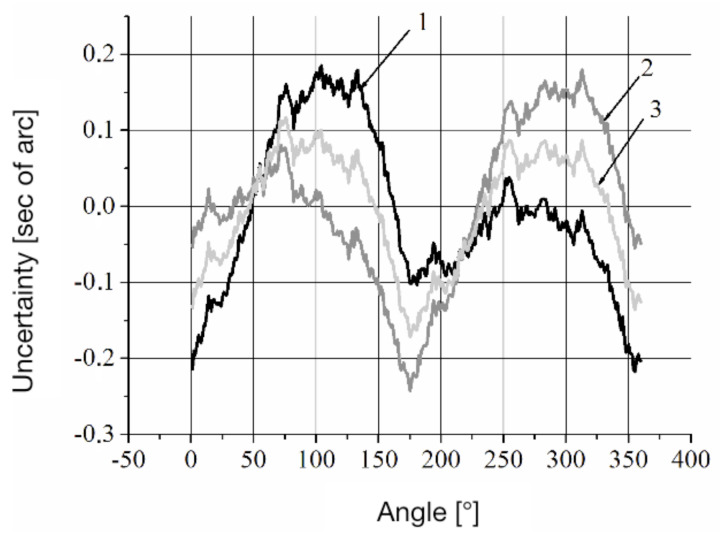
Illustration of the systematic error of the LDG: 1—RL CCW rotation; 2—RL CW rotation; 3—systematic error of the optical encoder.

The experimental LDG was built using a precision aero-static support and an optical encoder (OE) A-205. The OE A-205 does not have its own spindle and consists of a measuring scale (grating) that is installed on the spindle of the angle measurement system; two measurement heads with indicator grids and a zero-mark head are installed on the stator of the system. The principle of operation of the optical encoder is based on the modulation of the light flux by two diffraction gratings installed with a gap, thereby forming a two-beam interferometer in which one of the gratings moves relative to the other. The movable (measurement) grating has the form of a circular track of radial marks on a glass disk, which is fixed to the hub and mounted on the spindle of the system. The fixed (indicator) grating has the form of a fragment of a circular track of radial marks on a glass plate, which is built into the reading head fixed to the stator of the system. When the measurement grating rotates, the intensity of the light flux received by the photodiodes installed behind the indicator grating is modulated. The reading head has four photodiodes that produce four signals with relative phase shifts 0°, 90°, 180° and 270°. Phase shifts of the signals result from a small radial shift of the indicator grating, which leads to the formation of interference fringes of finite width, in the area where photodiodes are installed. The optical encoder has two identical reading heads installed, positioned diametrically opposite to the measuring disk axis of rotation. The value of the angular displacement of the measurement disk is determined by the average value calculated from the readings of the two measurement heads. The design of the optical encoder is conventional with a few extra features: the averages of the signals from two heads of the optical encoder are obtained algorithmically and not by means of hardware, as in the Heidenhain sensors, for example; the measurement grating has a small step size, namely, 162,000 marks are applied on a diameter of 108 mm. In this case, the number of periods of the sinusoidal signal on the output of the reading head is 324,000 per revolution of the spindle. The ring laser and optical encoder A-205 are rigidly fixed to the LDG spindle, which is rotated by a DC motor. The data acquisition from the optical encoder at angular intervals is set by software. The output signals of the optical encoder and the ring laser are passed through the corresponding signal converters and then sent to the electronics unit that pre-processes the data and transmits it to a personal computer. The measurement process was carried out over 25 revolutions of the LDG spindle, while data were taken from the ring laser at equal angular intervals, set by the optical encoder signals. Figure 3 shows the results of the measurement of the optical encoder. The measurements were performed when the ring laser rotated clockwise and counter-clockwise at a velocity of approximately 90°/s.

**Figure 4:**
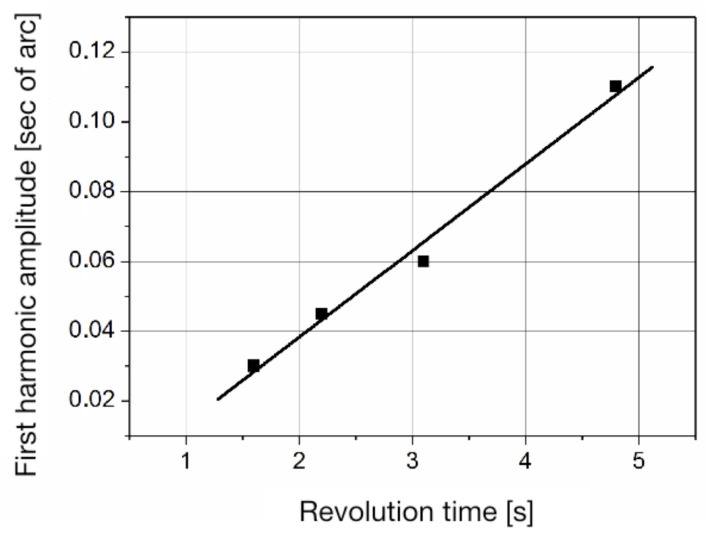
The dependence of the amplitude of the first harmonic on the revolution time.

Curve 1 corresponds to the systematic error of the LDG, defined by the expression (4), with the ring laser rotating counter-clockwise. Curve 2 illustrates the systematic error of the LDG, measured when the ring laser rotated clockwise, but reduced to a counter-clockwise rotation according to the expression (8). Curve 3 defines the systematic error of the optical encoder calculated by the Formula (13). The discrepancy between curves 1 and 2 is caused by a systematic error of ring laser, which can be found according to expression (12). Note that the amplitude of the systematic error of the ring laser at a constant external magnetic field depends on the rotation rate. Measurements taken by the reverse method allowed us to determine the dependence of the amplitude of the systematic error of the ring laser on the time of its rotation. This dependence is shown in Figure 4.

From Figure 4 it follows that the amplitude of the first harmonic of the systematic error of the ring laser linearly depends on the time of revolution.

### 6.2. Results of Optical Encoder Calibration in the “Off-Axis” Mode

**Figure 5:**
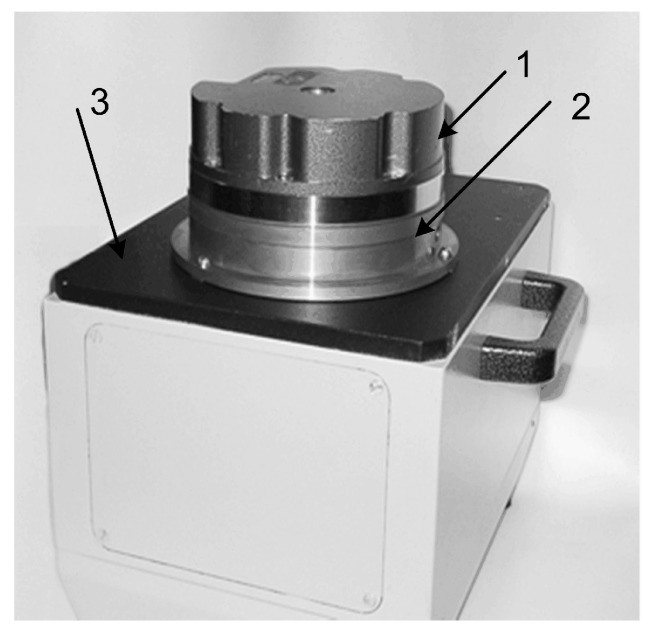
Installation of the LDG for the calibration of optical encoders; 1—RON-905; 2—adjustment ring; 3—LDG.

When installing the optical encoder on the LDG axis, the installation requirements set out in [9] are taken into account. The simplified cross-calibration method is used to eliminate the systematic error caused by errors in installing the optical encoder on the LDG axis. Let us discuss the results of the calibration of the optical encoder RON-905 of the company Heidenhain. The RON-905 has a built-in coupling and a closed hollow rotor, characterized by a systematic error not exceeding ±0.4 s of arc. To ensure reproducibility of the measurement results, the manufacturer Heidenhain sets hard requirements for its installation [15]. The setup of the LDG with the RON-905 installed on it is shown in Figure 5.

**Figure 6:**
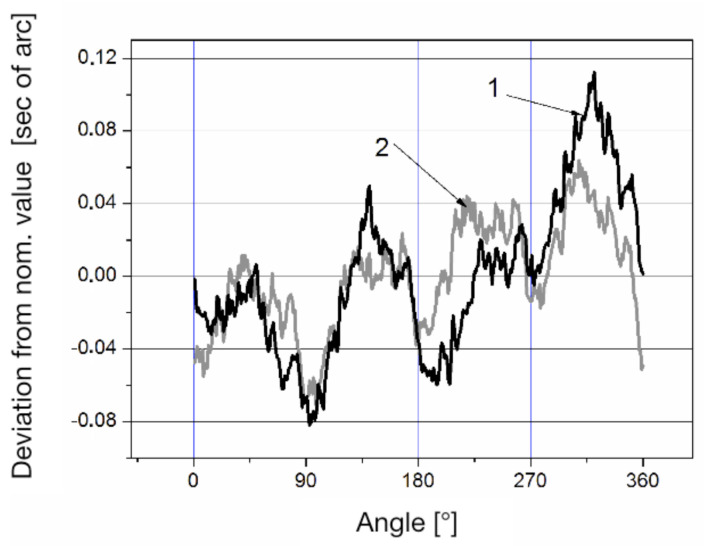
The results of the RON-905 calibration with rotation of the rotor in CW (2) and CCW direction (1).

The RON-905 stator (1) was mounted on a ring-shaped seat (2). The RON-905 rotor was connected to the axis of the LDG via an adapter made in accordance with the sensor installation requirements. A specially developed signal reading device was used to acquire data from the RL and RON-905, which provides a high acquisition rate and permits forming a reading signal from the RON-905 signals using a controlled frequency divider. Measurements were made for different directions of the rotation of the LDG rotor. The final results obtained for 12 positions of the RON-905 when the LDG rotor rotated clockwise and counter-clockwise are shown in Figure 6. These positions were chosen from a combination of three rotor positions 120° apart, for each of which there were four corresponding stator positions, 90° apart.

**Figure 7:**
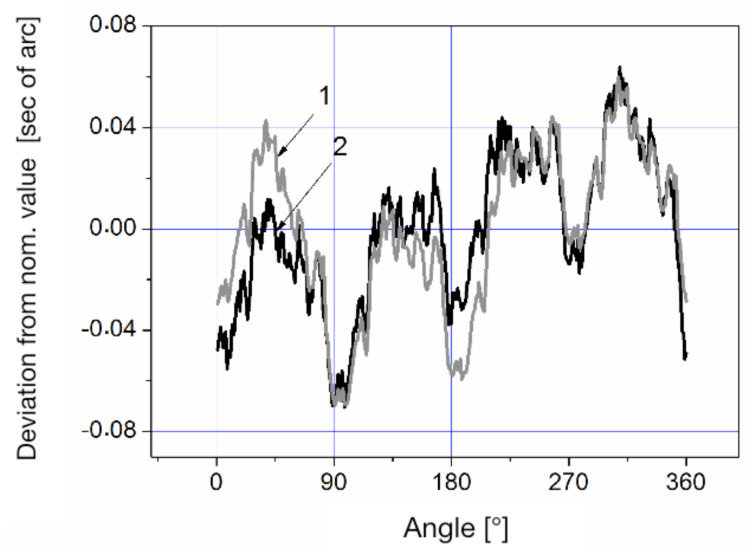
Results of the RON-905 calibration at the German National Metrological Institute (PTB) (1) and LDG (2).

The difference in the measurement results for the rotation in different directions is due to the influence of the magnetic field on the ring laser. Averaging these results yields the reduction of the error caused by the ring laser. Figure 7 shows a comparison of the RON-905 calibration results obtained on the LDG and at the “Physikalisch Technische Bundesanstalt” (PTB, Germany) on the type WMT-220 comparator.

The comparison of the two systems shows that the difference in calibration results does not exceed 0.03 s of arc.

## 7. The Development of High-Precision Theodolites and Total Stations Based on the LDG

When conducting research in the field of geodesy and geophysics, ring laser gyroscopes are widely used in the development and application of gyro-compassing techniques. This area is one of the fields where the advantages of a ring laser gyroscope are best demonstrated and the results are widely recognized [16,17]. However, some geodetic applications require contactless optical measurements of angles and directions involving precisely located prism reflectors or mirrors [18,19]. Such measurements usually are conducted with the help of theodolites or total stations. These devices are very convenient for conducting measurements, but the accuracy of their angle measurements is not very high. Usually they are limited to about one second of arc. In some applications the required accuracy is higher by one order of magnitude. The traditional way of reaching this high level of accuracy is by utilizing a turntable and a high-precision autocollimator with provisions for good accuracy of positioning and mounting on this platform. The turntable usually contains an optical angular encoder and the drive. The disadvantages of this technique are the long duration of the measurement procedure and the rather involved measurement process. This also carries the risk of additional non-stationary temperature effects. Another approach is the method of dynamical angle measurements [6]. In this method the ring laser continuously rotates around the vertical axes together with an optical null-indicator (NI). The null indicator generates pulses at those instants of time when the optical beam has normal incidence on the reflector. The intervals between these pulses define the angles between mirror surfaces. To measure these angles, one has to count the number of the ring laser output pulses falling inside the intervals. In that respect this procedure is very close to procedure of the LDG in the mode of optical polygon calibration. The main difference is the inverted procedure. Our measurement system was made on the basis of the ring laser GL-1 with a perimeter of 40 cm. The GL-1 is mounted on the shaft of the system. The rotation of the shaft is provided by a DC motor and the rotation rate is at the level 180°/s. On the upper part of the shaft, the turntable is mounted with the auto-collimating null indicator. The electronic unit of the signal preprocessing is also located on the turntable. The system operates in the following way. At the instants of the time when the beam of the null indicator falls normally onto the mirrors, it generates a pulse on the output of the null indicator. These pulses form the intervals over which the ring laser pulses are summed up. The shape of the output pulse of the null indicator is quasi-triangular. On the rising edge of the pulse, the preprocessing electronics form the TTL pulse, which starts the analog to digital conversion (ADC) and reads the counter of the ring laser output pulses. On the falling edge of the pulse the preprocessing electronics forms the TTL pulse, which stops the data acquisition. The ADC digitizes the analog output signal from the null indicator and the preprocessing electronics find the “center of gravity” of the output pulse of the null indicator. This information provides the additional part of the ring laser output signal, which is summed up with the counter information. The knowledge of the angular value of the ring laser output pulse provides the values of the measurement angles. Tests of the system were carried out when it was installed on the foundation to ensure automatic auto-collimation binding to four control elements (mirrors). Mirror control elements were placed around the circle on special foundations. The tests were performed in a series of measurements with the results displayed on the screen of the information processing and display unit. The obtained test results confirmed the possibility of auto-collimation sighting at a distance of at least 2 m on mirror control elements, and the ability of the developed system to measure horizontal angles in the range of 360 degrees. The following performance parameters were evaluated during the tests:

- The mathematical expectation, deviated from average and root mean square (RMS) of the measurement results of each individual angle obtained in a series of measurements.
- The absolute error of measurement of the sum of angles (the value of its deviation from 360°).

To assess the mathematical expectation (average) and RMS (internal convergence) of the measurement results, a series of measurements were performed, covering six measurement cycles, providing for consecutive measurements (in a circle) of the angles between the control mirrors. The results of measurements and estimates of their errors are shown in Table 2.

The differences between the sum of the values of the obtained angles and 360° are on the level of several hundredths of a second of arc, which is in excellent agreement with the expectations. Furthermore, the results obtained during the tests show that the random error of the angle measurement does not exceed 0.3 s of arc. Results of a system calibration performed by comparison with a reference optical polygon provide a systematic error at the level of 0.2 s of arc [20].

## 8. Discussion

Dynamic goniometry has been developed over 30 years. During this time the reference standards of angular measurements were created, which are used in various metrological institutes for calibration of optical polygons and for the transfer of the plane angle unit from the standard to the measuring instruments [21]. The reference standards for the reproduction of a flat angle unit in a dynamical environment were developed [12]. Utilizing the method of least deviation angle measurement [8], a reference setup was created for the measurement of the refractive index of solids. An important direction in the development of dynamic goniometry was the creation of test benches for monitoring the parameters of digital angle converters [12,22]. In recent years, the research area associated with the creation of systems for non-contact optical measurements of angles between some directions set in the space by prism reflectors or mirrors has begun to develop rapidly [20,23]. The application range of dynamic goniometry systems is expanding further, and at the same time their accuracy is improving. Today the accuracy of some goniometric systems can be resolved to the level of one hundredth of a second of arc. This level, however, is not considered particularly high today. A number of angular comparators (for example those at the PTB in Germany or INRIM in Italy) deliver an accuracy of several thousandths of an arc second. A further improvement in the accuracy of angular measurements requires an increased resolving power and improved long-term stability of the utilized ring lasers and optical encoders. Furthermore, an improvement of the means for the transfer of the angle unit would be required. How practical would such an improvement be? Since the sensitivity of a ring laser gyro can be improved by upscaling, a larger gyro provides a better resolution. If we compare our goniometer to the large square ring laser G, which was built with a length of 4 m on the side [24], we find an angular resolution of $\delta \varphi \approx 2\times 10^{-4}$ seconds of arc. In terms of size and weight, such a large scale structure would be entirely impractical. At that level of resolution, one would also have significant issues with errors due to sensor deformation and the stability of the orientation of the sensitive axis of the gyro. The 1 m${}^{2}$ C-II monolithic ring laser is already considerably smaller than G, but still weighs almost 700 kg. According to the operational parameters of C-II (see Table 2 in [24]) the resolution would be about one order of magnitude below G. A goniometer based on the square Russian KM-40 ring laser gyro has a length of 40 cm on the side and a weight of less than 30 kg. Operating with low-loss ion beam sputtered mirrors in order to make the quality factor of the cavity as large as possible would still provide a significant improvement as the figure of merit—namely, the ratio of the area over the perimeter would increase by a factor of four over the here reported instrumentation. This example illustrates how far the goniometry with active ring lasers has already come. Some improvement can be expected, but it will be less than an order of magnitude.

## Figures and Tables

**Table 1:** Here ${\bar{\omega }}_{i}$—average rotation rate of the ring laser (RL) during measurement $t_{i}$; $\varphi_{i}^{r}={\bar{\omega }}_{i}t_{i}$—angle of RL rotation during measurement $t_{i}$. *T* is the full revolution time of the RL in the laboratory reference system; ${\bar{\omega }}_{T}$—average rotation rate of the RL during the full revolution; $J_{i}=\int_{0}^{t_{i}}\;\frac{1}{\omega (t)}\;dt$, $J_{T}=\int_{0}^{T}\;\frac{1}{\omega (t)}\;dt$; the “+” or “−” sign is determined by the rotation direction of the RL—the “+” sign for CW and the “−” sign for the CCW.

Measurement Method	Measurement Equation	Methodic Uncertainty
phase	$\varphi =2\pi \frac{N_{i}}{N_{2\pi }}$	$\frac{K_{0}+K_{1}\Omega_{E}}{K_{1}}\frac{\left({\bar{\omega }}_{T}-{\bar{\omega }}_{i}\right)}{{\bar{\omega }}_{T}{\bar{\omega }}_{i}}\varphi_{i}^{r}+\frac{K_{-1}}{K_{1}}\left(J_{i}-J_{T}\frac{\varphi_{i}^{r}}{2\pi }\right)$
phase-time	$\varphi_{i}^{*}=2\pi \frac{N_{i}\pm N_{2\pi }\frac{\Omega_{E}t_{i}}{2\pi }}{N_{2\pi }\left(1\pm \frac{\Omega_{E}T}{2\pi }\right)}$	$\varphi_{i}^{r}\left(\frac{K_{0}}{K_{1}}\frac{\left({\bar{\omega }}_{T}-{\bar{\omega }}_{i}\right)}{{\bar{\omega }}_{T}{\bar{\omega }}_{i}}+\frac{K_{-1}}{K_{1}}\frac{\left(2\pi J_{i}-\varphi_{i}^{r}J_{T}\right)}{2\pi \varphi_{i}^{r}}\right)$
	$\varphi_{i}^{**}=2\pi \frac{N_{i}\pm Ft_{i}}{N_{2\pi }\pm FT}$	$\varphi_{i}^{r}\frac{K_{-1}}{K_{1}}\frac{\left(2\pi J_{i}-\varphi_{i}^{r}J_{T}\right)}{2\pi \varphi_{i}^{r}}$

**Table 2:** Deviation from the mean in units of seconds of arc.

1	−0.01	0.02	−0.07	0.06
2	0.02	−0.02	0.00	0.00
3	−0.05	0.04	−0.01	0.03
4	−0.02	−0.01	−0.03	0.06
5	0.00	−0.03	−0.11	−0.08
6	0.06	−0.02	0.01	−0.05
**mean value**	74°27′39″, 51	43°32′56″, 71	160°29′24″, 74	81°29′59″, 04
**RMS (s of arc)**	0.04	0.03	0.06	0.06
